# Tularemia following a squirrel bite in Southern Germany: a case report

**DOI:** 10.1007/s15010-025-02723-5

**Published:** 2026-01-24

**Authors:** Camilla Hahn, Elias Walter, Daniela Jacob, Eva-Maria Neurohr, Sabine Bélard

**Affiliations:** 1 Institute of Tropical Medicine, University of Tübingen, Wilhelmstr. 27, 72074 Tübingen, Germany; 2 German Center for Infection Research (DZIF), Tübingen, Germany; 3 Institute of Medical Microbiology and Hospital Hygiene, University Hospital Tübingen, Tübingen, Germany; 4 National Reference Laboratory for Francisella tularensis, Centre for Biological Threats and Special Pathogens, Highly Pathogenic Microorganisms (ZBS 2), Robert Koch Institute, Berlin, Germany

**Keywords:** Tularemia, Squirrel bite, Emerging zoonotic infections, Wildlife exposure, Endemic regions

## Abstract

**Background:**

Tularemia is a zoonotic infection caused by *Francisella tularensis*. Human cases in Germany are emerging and are usually associated with hare or tick exposure. Transmission via squirrel bites has only been reported once before in Germany.

**Case presentation:**

We describe a 23-year-old woman who developed tularemia 11 days after being bitten by an injured red squirrel (*Sciurus vulgaris*) in southern Germany. Initial symptoms included headache, chills and fatigue, followed by painful right axillary lymphadenopathy without fever. Empirical ciprofloxacin (500 mg twice daily) was initiated on day 10 post-bite. *Francisella* *tularensis* serology was negative at presentation but seroconversion was documented 19 days later, confirming the diagnosis. The patient recovered fully after a 10-day course of ciprofloxacin.

**Conclusions:**

This case underscores the need to consider tularemia after rodent bites in endemic areas, including squirrel bites, and highlights the favourable clinical course following early fluoroquinolone therapy.

## Introduction

Tularemia, caused by the Gram-negative coccobacillus *Francisella tularensis*, is an emerging zoonosis in Europe [1–3]. In Germany, the annual incidence is low but rising in recent years, with 2.6 cases/1 million population in 2024 [1]. Most infections are linked to contact with hares, ticks or contaminated water or dust. Ulceroglandular tularemia, the commonest clinical form, follows inoculation through the skin and is characterized by a local ulcer and regional lymphadenitis. Squirrel-associated transmission of tularemia has been described only once before in Germany [4]. We report a case of tularemia after a squirrel bite to raise awareness of this unusual transmission route.

## Case presentation

A 23-year-old previously healthy woman was referred to the outpatient Clinic for Tropical and Travel Medicine, University Hospital Tübingen, in summer 2024. Eleven days earlier, within the local area of Baden-Württemberg, she had found an immobile red squirrel and had attempted to pick it up. The squirrel had bitten the distal phalanx of her right middle finger, causing a puncture wound. The patient had cleansed the wound with povidone-iodine ointment (Betaisodona®) and had received a tetanus booster from her general practitioner (GP).

Seven days post-bite, she developed headache, chills and fatigue without measured fever. Another three days later, she noted a painful swelling in the right axilla and inner upper arm. Suspecting tularemia, her GP started oral ciprofloxacin 500 mg twice daily on day 10 post-bite and referred her for diagnostic confirmation.

At presentation for the diagnostic confirmation (day 11) the patient reported persistent headache and fatigue. Vital signs were normal, and the patient was afebrile and in good condition. A small, tender, mobile lymph node was palpable in the right axilla. A pressure-sensitive area on the inner side of the distal right upper arm was noted without palpable induration. The distal phalanx of the right third digit displayed mild apical erythema and swelling at the site of the bite wound; the skin was closed.

Past medical history was unremarkable except for an episode of herpes zoster one year earlier. The patient denied any history of tick bites but reported frequent mosquito bites. She had regular contact with horses and dogs. Baseline laboratory tests, including complete blood count, C-reactive protein, liver and renal function parameters, were normal besides mild anaemia (hemoglobin 11.2 g/dl, reference 12.0–16.0 g/dl). Serological detection of antibodies against *F. tularensis* was inconclusive (IgG 14.4 U/ml (borderline positivity) and IgM < 10.0 U/ml (negative)). Further diagnostics like biopsy of the lymph node or wound swab were not performed as the skin was closed on the day of presentation making sampling of material for PCR more traumatic.

At follow-up 29 days after the bite (19 days after initial presentation and following completion of a 10-day course of ciprofloxacin), the patient was asymptomatic. She reported that the axillary lymphadenopathy had resolved within five days and headaches within three days after starting antibiotic treatment. The bite site had healed, leaving only mildly thickened skin. Repeat serology showed a significant rise in antibody titre with *F. tularensis* specific antibodies IgG 44.1 U/ml and IgM 224.7 U/ml, confirming seroconversion. Serological testing of both baseline and follow-up serum by the National Reference Laboratory for *F. tularensis* confirmed seroconversion (baseline IgM and IgG negative, follow-up IgM 1:4000 and IgG 1:1000; ELISA detecting antibodies against the lipopolysaccharide of *F. tularensis*). Seroconversion thus confirmed clinical diagnosis of tularemia. As only serological evidence was available, subspecies differentiation was not possible. Considering that the squirrel bite occurred in Germany, where no human infections with *F. tularensis* ssp. *tularensis* have been reported and yet only ssp. *tularensis* and ssp. *holarctica* are of clinical relevance, the infection was most likely due to *F. tularensis* ssp. *holarctica*. Confirmation was not feasible, as no material was available for molecular analysis.

## Discussion

Tularemia is an emerging zoonotic disease in Germany, with most human infections associated with contact with lagomorphs, tick bites, or contaminated water or dust [2, 3]. Human infections from squirrels are extremely uncommon, with this case representing one of only two published instances of confirmed *Francisella tularensis* transmission following a squirrel bite in Germany [4]. Other few cases of tularemia transmitted by squirrels have been reported from the United States [5]. In 2021 natural infection of a squirrel with *F. tularensis* ssp. *holarctica* was reported from Switzerland [6]. In Germany, mandatory reporting of tularemia in animals applies only to hares and rabbits. The national reference institute for animal health, Friedrich Loeffler Institut, and its regional partner in Baden-Württemberg never received a squirrel carcass for tularemia confirmation. Moreover in a study assessing the prevalence of tularemia among wild animals in Germany, squirrels were not included as a surveyed species [7].

In contrast to the unknown prevalence of tularemia in squirrels in Germany, recent surveillance data from Baden-Württemberg, where our case occurred, reveals important overall epidemiological trends that contextualize this unusual transmission route [8]. During 2012–2022, Baden-Württemberg reported 152 human tularaemia cases with a significant 20% annual increase, peaking at 35 cases in 2021 [8]. This figure was exceeded in 2024 with 51 human cases and represents one of the highest incidence rates among German federal states, with 4.5 cases per million population in 2024, substantially exceeding the national average of 2.6 cases per million [1].

Our case, along with the previous Bavarian squirrel bite case [4], suggests that the reservoir spectrum for *F. tularensis* in Germany may be broader than traditionally recognized. While brown hares remain the known reservoir host, surveillance data from Baden-Württemberg indicates *F. tularensis* detection in various other animals, including wild boar, rodents, domestic animals, and notably, an Eurasian blue tit [8]. Thereby, this case underscores the need for broad awareness of tularemia transmission risks beyond traditional reservoirs and with negative infection markers as C-reactive protein was negative on day 11 post infection and only one day of treatment.

While brown hares remain the primary concern, the public's propensity to handle distressed wildlife, particularly urban-adapted species, may represent an underrecognized transmission route [9]. Healthcare providers should maintain a high index of suspicion for tularemia following any wildlife contact, regardless of species, particularly in endemic regions like Baden-Württemberg. The recent publication by Nothdurfter et al. [8] shows that arthropod bites, including tick bites, are the most often exposure mentioned in combination with an infection in Baden-Württemberg. Arthropods, especially ticks in Germany, can serve as vectors for transmitting the disease between species.

In general, for diagnostics using specific antibodies against *F. tularensis*, it is important to note that these become detectable approximately three weeks after exposure or two weeks after the onset of symptoms. This depends on the method and assay used (commercial or in-house). The time of seroconversion also depends, among other things, on the infection dose [10]. Interestingly, IgM- and IgG- antibodies increase almost in parallel and it is known that all isotypes (IgM, IgG and IgA) can persist for years [11].

If tularemia is suspected, even at an early stage, it is advisable to take further samples such as blood cultures, wound swabs or lymph node biopsies in order to confirm the diagnosis as quickly as possible in the laboratory. Antibiotic therapy should be started as soon as possible with antibiotics like ciprofloxacin or doxycycline as recommended by the German working group on highly pathogenic pathogens (STAKOB) [10].

In conclusion, while this squirrel-associated case represents an unusual transmission route within the German epidemiological context, it occurs against a background of increasing tularemia incidence and evolving transmission pattern in Southern Germany. This case contributes to expanding our understanding of potential *F. tularensis* reservoirs and emphasizes the importance of considering tularemia in any wildlife exposure scenario within endemic regions.

## Data Availability

No datasets were generated or analysed during the current study.
